# Drug-Induced Hyperglycemia as a Potential Contributor to Translational Failure of Uncompetitive NMDA Receptor Antagonists

**DOI:** 10.1523/ENEURO.0346-21.2021

**Published:** 2021-12-21

**Authors:** Eric Yuhsiang Wang, Ted Weita Lai

**Affiliations:** 1 Graduate Institute of Biomedical Sciences, China Medical University, Taichung 404, Taiwan; 2 School of Medicine, China Medical University, Taichung 404, Taiwan; 3 Drug Development Center, China Medical University, Taichung 404, Taiwan; 4 Translational Medicine Research Center, China Medical University Hospital, Taichung 404, Taiwan

**Keywords:** circadian, excitotoxicity, hyperglycemia, neuroprotection, NMDA receptor, stroke

## Abstract

Hyperglycemia is a comorbidity in 60–80% of stroke patients; nevertheless, neuroprotective drugs like NMDA receptor (NMDAR) antagonists are typically assessed in normoglycemic animals at the preclinical stage before they are approved to enter clinical trials. Interestingly, as a possible explanation for the translational failure of NMDAR antagonists, it was recently reported that stroke occurring during nighttime causes smaller infarctions in rodents and therefore has a smaller window for neuroprotection. To investigate why stroke occurring during different circadian phases confers a difference in severity, we reanalyzed the published source data and found that some mice that were used in the daytime have higher blood glucose than mice that were used in the nighttime. We then repeated the experiments but found no difference in blood glucose concentration or infarct volume regardless of the circadian phase during which stroke occurs. On the other hand, induction of hyperglycemia by glucose injection reproducibly increased stroke severity. Moreover, although hyperglycemia increases infarction volume, which presumably would provide a larger window for neuroprotection, uncompetitive NMDAR antagonists were unexpectedly found to exacerbate stroke outcome by worsening hyperglycemia. Taken together, our new data and reanalysis of the published source data suggested that blood glucose during stroke, rather than the circadian phase during which stroke occurs, affects the size of the ischemic infarction; moreover, we have revealed drug-induced hyperglycemia as a potential reason for the translational failure of uncompetitive NMDAR antagonists. Future trials for this class of neuroprotective drugs should monitor patients’ blood glucose at enrollment and exclude hyperglycemic patients.

## Significance Statement

Hyperglycemia is highly prevalent among stroke patients (60–80%; Scott et al., 1999), and blood glucose is a chief determinant of stroke severity in the clinic. Nevertheless, preclinical stroke studies were typically performed using normoglycemic animals. Here, we reported evidence that uncompetitive NMDA receptor (NMDAR) antagonists, a major class of neuroprotective drugs, can exacerbate stroke outcome in hyperglycemic and diabetic mice. Our study revealed a previously unknown explanation for the translational failure of NMDAR antagonists, thereby providing new guidance for the future design of clinical stroke trials. In addition, this report serves as a case for the need to assess neuroprotective drugs in hyperglycemic animals before they should be approved to treat stroke patients in clinical trials.

## Introduction

Despite the success of NMDA receptor (NMDAR) antagonism in some animal stroke models, NMDAR antagonists and NA1, a therapeutic peptide designed to minimize NMDAR-mediated free radical damage, have failed to demonstrate unequivocal clinical benefit in human stroke trials (Lai et al., 2014; Hill et al., 2020). Of the potential explanations for why inhibition of NMDARs or their release of free radicals has failed to translate from bench to clinic, several studies have explored the possibility that these therapeutic agents are neuroprotective only when certain experimental or clinical conditions are met. For example, two recent studies have supported the notion that these neuroprotective agents, including NMDAR antagonists and NA1, are effective only in animals subjected to middle cerebral arterial occlusion (MCAO), which produces a large hemispheric infarct, but not in animals subjected to distal MCAO (dMCAO), which produces a smaller localized cortical infarct (Liu et al., 2020, 2021). These findings complement previous reports showing that neuroprotection by MK801, a prototypical NMDAR antagonist, strongly depends on the type of cerebral ischemia that is used to assess drug efficacy (Buchan et al., 1991; LeBlanc et al., 1991; Nellgård et al., 1991; Nellgård and Wieloch, 1992). Moreover, even in the same stroke model, the neuroprotective efficacy of MK801 has been widely reported to be contingent on the body temperature of the animal during the experiment (Ikonomidou et al., 1989; Buchan and Pulsinelli, 1990; Corbett et al., 1990; Warner et al., 1991; Memezawa et al., 1995; Alkan et al., 2001; Gerriets et al., 2003).

Recently, one prominent study has added to the flurry of experimental evidence showing that neuroprotection by MK801 or α-phenyl-butyl-tert-nitrone, a scavenger that can prevent NMDAR-dependent free radical release, is contingent on the conditions under which stroke occurs (Esposito et al., 2020). In particular, the authors report that these therapeutic agents are neuroprotective only in rats and mice when stroke is induced during the daytime (rodent sleep cycle) but not when stroke is induced during the nighttime (rodent wake cycle). Given that rats and mice are nocturnal animals, the authors suggested that the opposite phenomenon would be true in human stroke patients. Because human subjects enrolled in clinical trials mostly experience stroke during the daytime (human wake cycle), the findings of that study could have important implications for how stroke trials should be redesigned to properly assess the potential clinical benefits of these treatments. Furthermore, to explain why these neuroprotective agents are effective only during the daytime, the authors compared cortical infarction between animals in which stroke was induced during the daytime and those in which stroke was induced during the nighttime and found that cortical infarction, spread of the penumbra, and density of neurodegeneration were much more severe when stroke was induced during the daytime than when it was induced during the nighttime. Based on these findings, the authors suggested that there might be more room for neuroprotection when stroke occurs during the daytime (Esposito et al., 2020).

In light of a lack of *in vivo* data to explain the larger cerebral infarction in the daytime group in that study (Esposito et al., 2020), we reanalyzed the published source data on animal physiology from that study to check whether animals that underwent surgery in the daytime had higher blood glucose concentrations. As decades of clinical and experimental evidence have already established blood glucose to be a key factor in determining stroke severity in human patients and laboratory animals, we next asked whether exacerbating ischemic brain infarction by increasing blood glucose would unmask a neuroprotective effect of NMDAR antagonists in a stroke model that is otherwise resistant to protection by these drugs. Unexpectedly, we found that the opposite is true. Surprisingly, uncompetitive antagonists of NMDARs can further increase blood glucose in hyperglycemic animals, thereby exacerbating their stroke outcome.

## Materials and Methods

### Mice and cerebral ischemia

Adult male mice (C57BL/6; 7–10 weeks old; 21–30 g) were purchased from the National Laboratory Animal Center (Taipei, Taiwan), housed in our institutional animal care facility under a 12/12 h light/dark cycle and given free access to food and water. All study protocols were conducted in accordance with the Institutional Guidelines of the China Medical University for the Care and Use of Experimental Animals (IGCMU-CUEA) and were approved by the Institutional Animal Care and Use Committee of the China Medical University (IACUC-CMU; Taichung, Taiwan; Protocol no. CMUIACUC-2021-278). Cerebral ischemia was induced in these mice by transient dMCAO under brief isoflurane anesthesia as described previously (Liu et al., 2017). Each mouse was subjected to a 2-h occlusion period, during which the mouse was kept normothermic by means of an autoregulated heating pad with continuous feedback from a rectal temperature probe. After surgery, the mice were allowed to recover in their home cage, where they remained until killing by urethane overdose and perfusion with saline 24 h postischemia for collection of their brains for assessment of the infarction volume.

### Induction of acute hyperglycemia

To produce a hyperglycemic state in the mice during stroke, mice received an injection of saline (vehicle control, i.p.) or 30% glucose in saline (2.2 g/kg, i.p.) 20 min before the induction of cerebral ischemia by dMCAO; this injection typically resulted in a blood glucose concentration between 200 and 400 mg/dl before stroke onset. At the times specified in the manuscript, blood was collected from the severed tail, and blood glucose concentration was measured by a standardized glucometer (FORA, TD4272A).

### Induction of type 1 diabetes mellitus

To induce diabetes mellitus in mice before stroke, mice received repeated injections of freshly prepared streptozotocin (STZ; 40 mg/kg/d, i.p.), dissolved in citrate buffer (50 mm, pH 4.5), for five consecutive days. This protocol induced diabetes mellitus, as confirmed by hyperglycemia (blood glucose > 200 mg/dl) determined 8 d after the final dose of STZ, in 18 out of 22 mice. Those 18 diabetic mice were subjected to dMCAO 9 d after the final dose of STZ, and the four mice without diabetes were excluded without further experimentation.

### Quantification of brain infarction

Isolated mouse brains were coronally sectioned at a thickness of 1 mm on a brain matrix, and each coronal section was bathed in 2% 2,3,5-triphenyltetrazolium (TTC; Sigma-Aldrich) at 37°C for 10 min. The infarct and non-infarct areas of each coronal section were measured using the image analysis software ImageJ (NIH). Infarct volume was determined by multiplying the ipsilateral infarct area by the thickness of each coronal section. To account for potential edema in ischemic infarct, we also calculated a “normalized infarct volume corrected for edema” determined by multiplying normalized infarct area, calculated by subtracting ipsilateral non-infarct area from contralateral non-infarct area, by the thickness of each coronal section.

### NMDAR antagonists

MK801 (Abcam, catalog #ab120027), ketamine (Sigma-Aldrich, catalog #K2753-1G), D-2-amino-5-phosphonovaleric acid (AP5; Cayman, catalog #14539), and 3-(2-carboxypiperazin-4-yl)propyl-1-phosphonic acid (CPP; Sigma-Aldrich, catalog #C104-5MG) were used in this study. For mice subjected to cerebral ischemia, drugs were administered 60 min before ischemia onset.

### Randomization and exclusion

The investigators performing the surgery and quantification of brain infarction were blinded to the drug treatments until the completion of data analysis. As described above, four mice were excluded before surgery because STZ failed to induce diabetes mellitus (blood glucose > 200 mg/dl) in those mice. In addition, the stroke surgery had a failure rate of 10.6%: 13 out of 123 mice were excluded because the mice died during surgery or soon after reperfusion while investigators were still blinded to the drug treatments (Table 4). The excluded mice were evenly distributed across experimental groups, and accounted for the differences in sample sizes between blood glucose concentrations data and infarction volumes data. As shown in Table 4, except for the four mice excluded because of failure to induce diabetes, the excluded mice had blood glucose concentrations similar to non-excluded mice. No data collected were excluded from analysis.

### Data presentation and analysis

The data are presented as individual data points, with the error bars showing the mean ± SEM. Differences between two groups were compared by unpaired *t* test, and differences between multiple groups were compared by one-way or two-way ANOVA followed by Tukey’s multiple comparisons test. Changes in glucose concentrations were compared by two-way repeated-measures ANOVA matching blood glucose from the same animal, which was followed by Tukey’s multiple comparisons test.

## Results

### Effect of blood glucose and circadian time of stroke on infarction volume

**Figure 1.**
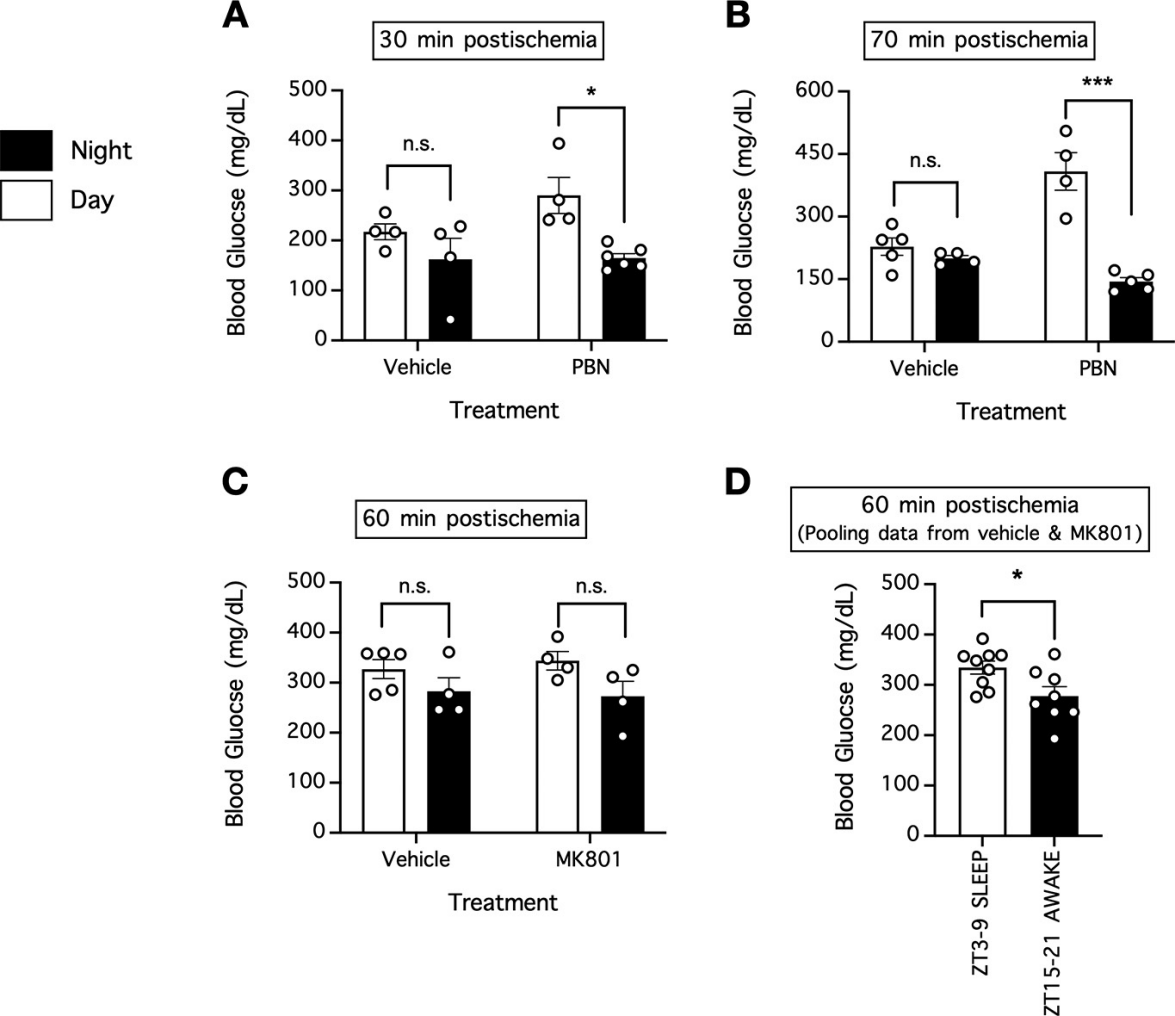
Blood glucose concentrations of the mice in the daytime groups versus the nighttime groups in the circadian stroke study (Esposito et al., 2020). ***A–C***, We reanalyzed the source data to compare the blood glucose concentrations of mice in the daytime groups versus the nighttime groups published in that study. Blood was collected at 30 min (***A***), 70 min (***B***), or 60 min (***C***) postischemia. In ***A–C***, *n* = 4–6 per group; *p < 0.05, ***p < 0.01, two-way ANOVA followed by Tukey’s multiple comparisons test; n.s. indicates no significant difference. ***D***, To increase statistical power for ***C***, we pulled the data from mice regardless of the treatment received and instead grouped the mice in accordance with phase of the circadian cycle. In ***D***, *n* = 8–9 per group; *p < 0.05, Student’s *t* test. PBN, α-phenyl-butyl-tert-nitrone.

The source data and data table published in the circadian study showed no difference between daytime and nighttime in terms of blood pH, blood pCO_2_, blood pO_2_, blood pressure, and body temperature (*p* > 0.05, Tukey’s multiple comparisons test; Table 1; Esposito et al., 2020), all of which are potential factors that can contribute to a difference in stroke severity. Moreover, the source data and data table presented the blood glucose concentrations of the mice used in that study, including their blood glucose concentrations at 30 min, 60 min, 70 min, and 24 h after cerebral ischemia, which was induced either during the daytime or nighttime and was with or without neuroprotective treatments (Esposito et al., 2020). Although the original report stated that blood glucose concentrations were similar across groups (Esposito et al., 2020), we found that the mice in the daytime [zeitgeber time (ZT)3–ZT9; sleep cycle for mice] groups tended to have higher blood glucose concentrations than the mice in the nighttime (ZT15–ZT21; awake cycle for mice) groups (Fig. 1*A–D*; Table 1). However, the difference was only statistically significant in drug-treated mice at 30 min (*p* = 0.0155, Tukey’s multiple comparisons test) and 70 min postischemia (*p* < 0.0001, Tukey’s multiple comparisons test), but not for either vehicle (*p* = 0.5424, Tukey’s multiple comparisons test) or drug-treated mice (*p* = 0.2161, Tukey’s multiple comparisons test) at 60 min postischemia (Fig. 1*A–C*; Table 1). For 60 min postischemia, we attempted to increase statistical power by pooling the blood glucose concentration data obtained in the daytime group and nighttime group, regardless of treatment received, and found that mice in the daytime group pooled together had higher blood glucose concentrations (334 ± 13 mg/dl; *n* = 9) than mice in the nighttime group pooled together (blood glucose of 278 ± 19 mg/dl; *n* = 8; *p* = 0.0223, *t* test; Fig. 1*D*; Table 1). We did not analyze the 24 h blood glucose data because the source data for that was incomplete. Taken together, these analyses showed that hyperglycemia in the mice of the daytime group could explain the larger observed cerebral infarction in that study.

**Table 1:** Statistical analysis of source data reported by Esposito et al. (2020)

30 min postischemia										
		Blood pH			pCO_2_			pO_2_		
		*N*	Mean ± SEM	*p* value	*N*	Mean ± SEM	*p* value	*N*	Mean ± SEM	*p* value
Vehicle	ZT3–ZT9	4	7.34 ± 0.01	0.6008^2^	4	39 ± 2	0.3587	4	193 ± 22	0.9176
	ZT15–ZT21	4	7.27 ± 0.04		4	47 ± 5		4	178 ± 12	
PBN^1^	ZT3–ZT9	4	7.29 ± 0.07	0.1949	4	41 ± 4	0.6195	4	155 ± 12	0.5174
	ZT15–ZT21	6	7.39 ± 0.003		6	35 ± 1		6	186 ± 15	
		Blood glucose			Blood pressure			Body temperature		
		*N*	Mean ± SEM	*p* value	*N*	Mean ± SEM	*p* value	*N*	Mean ± SEM	*p* value
Vehicle	ZT3–ZT9	4	217 ± 16	0.5101	4	92 ± 5	0.9059	4	37.1 ± 0.2	0.9880
	ZT15–ZT21	4	162 ± 42		3	88 ± 4		4	37.1 ± 0.2	
PBN	ZT3–ZT9	4	290 ± 36	0.0155	3	88 ± 4	0.9895	4	37.4 ± 0.04	0.9789
	ZT15–ZT21	6	165 ± 9		6	87 ± 3		6	37.3 ± 0.1	
60 min postischemia										
		Blood pH			pCO_2_			pO_2_		
		*N*	Mean ± SEM	*p* value	*N*	Mean ± SEM	*p* value	*N*	Mean ± SEM	*p* value
Vehicle	ZT3–ZT9	5	7.35 ± 0.02	0.1124	5	36 ± 2	0.8346	5	170 ± 6	0.9944
	ZT15–ZT21	4	7.40 ± 0.01		4	33 ± 2		4	167 ± 8	
MK801	ZT3–ZT9	4	7.29 ± 0.02	0.3365	4	40 ± 2	0.9996	4	171 ± 8	0.4415
	ZT15–ZT21	4	7.33 ± 0.004		4	40 ± 1		4	154 ± 9	
		Blood glucose			Blood glucose (pooled)			Blood pressure		
		*N*	Mean ± SEM	*p* value	*N*	Mean ± SEM	*p* value	*N*	Mean ± SEM	*p* value
Vehicle	ZT3–ZT9	5	327 ± 19	0.5424	ZT3–ZT9 all mice		0.0223 *t* test	5	85 ± 2	0.9997
	ZT15–ZT21	4	283 ± 27		9	334 ± 13		4	85 ± 2	
MK801	ZT3–ZT9	4	344 ± 18	0.2161	ZT15–ZT21 all mice			4	84 ± 0.3	0.9823
	ZT15–ZT21	4	273 ± 30		8	278 ± 19		4	85 ± 2	
70 min postischemia										
		Blood pH			pCO_2_			pO_2_		
		*N*	Mean ± SEM	*p* value	*N*	Mean ± SEM	*p* value	*N*	Mean ± SEM	*p* value
Vehicle	ZT3–ZT9	5	7.31 ± 0.01	0.9952	5	43 ± 1	0.9943	5	200 ± 23	0.6341
	ZT15–ZT21	4	7.32 ± 0.03		4	44 ± 4		4	165 ± 27	
PBN	ZT3–ZT9	4	7.18 ± 0.08	0.1682	4	45 ± 5	0.4566	4	155 ± 16	0.7998
	ZT15–ZT21	4	7.32 ± 0.01		4	38 ± 1		4	184 ± 15	
		Blood glucose			Blood pressure			Body temperature		
		*N*	Mean ± SEM	*p* value	*N*	Mean ± SEM	*p* value	*N*	Mean ± SEM	*p* value
Vehicle	ZT3–ZT9	5	227 ± 21	0.8574	5	87 ± 3	0.9859	5	37.3 ± 0.1	0.8177
	ZT15–ZT21	4	200 ± 7		3	86 ± 2		4	37.5 ± 0.1	
PBN	ZT3–ZT9	4	408 ± 45	<0.0001	4	82 ± 3	0.9045	4	37.4 ± 0.2	0.8293
	ZT15–ZT21	4	144 ± 13		4	80 ± 3		4	37.6 ± 0.2	

1 PBN, α-phenyl-butyl-tert-nitrone.

2 Unless otherwise mentioned, analyses were done by two-way ANOVA followed by Tukey’s multiple comparisons test.

**Figure 2.**
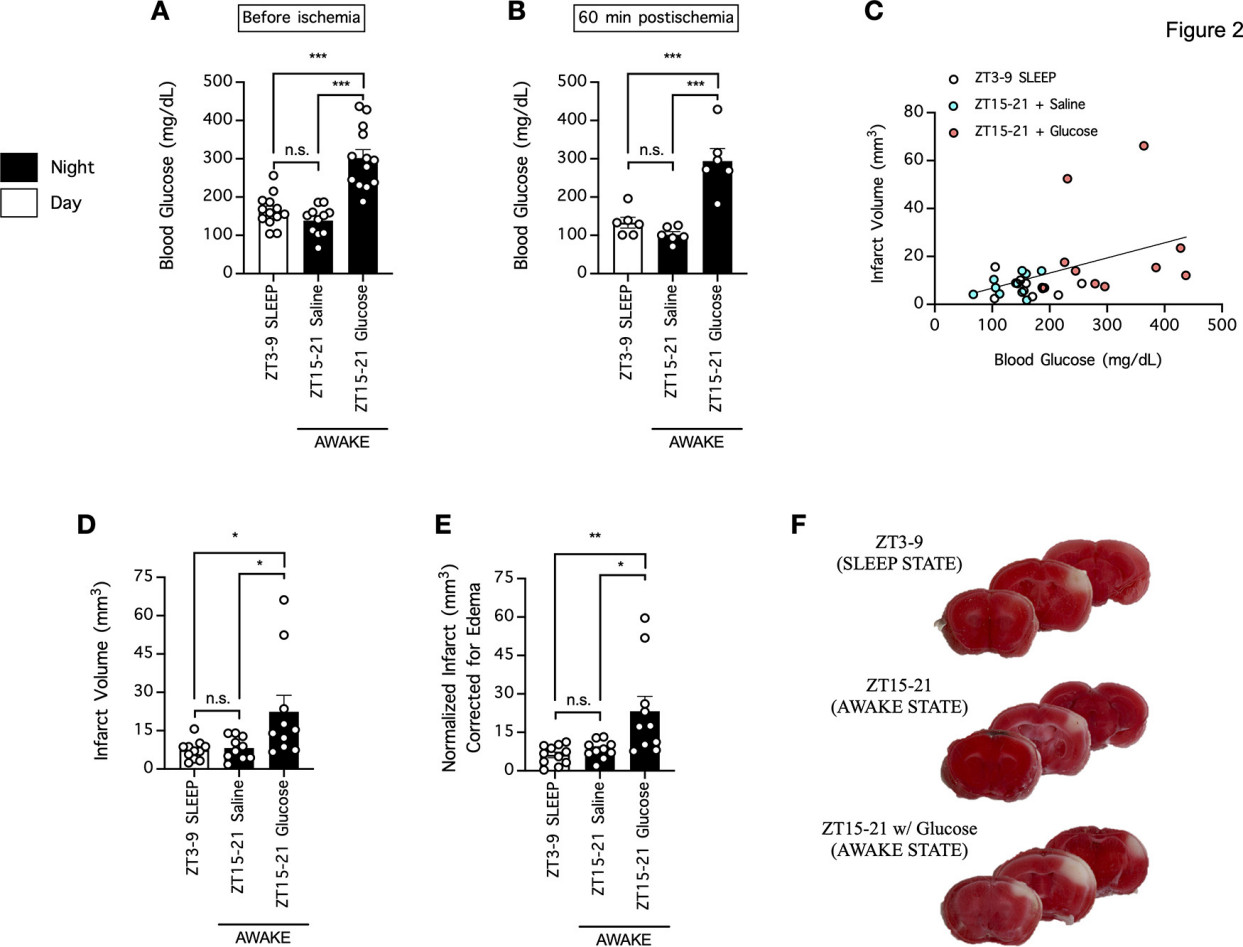
Hyperglycemia rather than the circadian phase during which stroke occurs determines the ischemic infarction volume. ***A***, Blood was collected to measure blood glucose concentrations from mice in the daytime group (ZT3–ZT9; sleep cycle for mice) or the nighttime group (ZT15–ZT21; awake cycle for mice), the latter group received either a saline or glucose injection (2.2 g/kg, i.p.) 20 min before blood collection; *n* = 11–13 per group. ***B***, Blood was also collected 60 min postischemia in a subset of mice in ***A***; *n* = 6 per group. In ***A***, ***B***, ***p < 0.001, one-way ANOVA followed by Tukey’s multiple comparisons test; n.s. indicates no significant difference. ***C***, ***D***, The mice from ***A*** underwent cerebral ischemia induction by dMCAO immediately following blood collection, and the ischemic infarction volume was assessed 24 h postischemia. Data were analyzed by linear regression (***C***) or one-way ANOVA followed by Tukey’s multiple comparisons test (***D***). ***E***, Same as ***D***, except infarct was normalized to account for potential brain edema. In ***D***, ***E***, *n* = 10–11 per group; *p < 0.05, **p < 0.01, one-way ANOVA followed by Tukey’s multiple comparisons test; n.s. indicates no significant difference. ***F***, Representative images of coronal brain sections of mice from ***C***, ***D***. Functional brain tissue was stained red by TTC, and the infarct area remained pale white and unstained.

To further explore this phenomenon, we collected blood from mice during the daytime (ZT3–ZT9; sleep cycle for mice) and nighttime (ZT15–ZT21; awake cycle for mice), with or without acute hyperglycemia induced by glucose infusion, and subjected the mice to cerebral ischemia by dMCAO thereafter to reassess the effect of the circadian rhythm and blood glucose on ischemic infarction (Fig. 2*A–F*; Table 2). In the present study, mice in the daytime and nighttime groups had blood glucose concentrations of 165 ± 12 mg/dl (*n* = 13) and 139 ± 11 mg/dl (*n* = 11; *p* = 0.5226, daytime compared with nighttime, Tukey’s multiple comparisons test), and mice that received a glucose infusion 20 min before blood collection had a blood glucose concentration of 302 ± 22 mg/dl (*n* = 13; *p* < 0.0001, glucose compared with no glucose, Tukey’s multiple comparisons test; Fig. 2*A*; Table 2). In a subset of mice, we also determined blood glucose 60 min postischemia, and found that mice in the daytime and nighttime groups had blood glucose concentrations of 133 ± 14 mg/dl (*n* = 6) and 101 ± 8 mg/dl (*n* = 6; *p* = 0.5515, daytime compared with nighttime, Tukey’s multiple comparisons test), and mice that received a glucose infusion 20 min before ischemia had a blood glucose concentration of 294 ± 33 mg/dl (*n* = 6) 60 min postischemia (*p* < 0.0001, glucose compared with no glucose, Tukey’s multiple comparisons test; Fig. 2*B*; Table 2). In light of overwhelming evidence for positive correlation between blood glucose and stroke severity in the clinic (Riddle and Hart, 1982; Pulsinelli et al., 1983; Candelise et al., 1985; Berger and Hakim, 1986; Weir et al., 1997; Capes et al., 2001; Williams et al., 2002), we did not observe a significant correlation between blood glucose of individual mice and their infarct volumes when each group of mice was assessed individually (*p* = 0.5332 for mice in the daytime group; *p* = 0.2075 for mice in the nighttime group; *p* = 0.7632 for mice with glucose pretreatment), but did find marginal yet significant correlation between blood glucose of individual mice and their infarct volumes when the data from all mice were pooled (*r*^2^=0.2004 and *p* = 0.0116; Fig. 2*C*; Table 3). With Tukey’s multiple comparisons test, we found that mice in the daytime and nighttime groups had infarction volumes of 7 ± 1 mm^3^ (*n* = 11) and 8 ± 1 mm^3^ (*n* = 10; *p* = 0.9851, daytime compared with nighttime), and mice that received a glucose infusion 20 min before cerebral ischemia had an infarction volume of 22 ± 6 mm^3^ (*n* = 10; *p* = 0.0352, glucose compared with no glucose; Fig. 2*D*,*F*; Table 2). When the infarct volume were normalized to account for potential cerebral edema, mice in the daytime and nighttime groups had infarction volumes of 6 ± 1 mm^3^ (*n* = 11) and 8 ± 1 mm^3^ (*n* = 10; *p* = 0.8985, daytime compared with nighttime), and mice that received a glucose infusion 20 min before cerebral ischemia had an infarction volume of 23 ± 6 mm^3^ (*n* = 10; *p* = 0.0116, glucose compared with no glucose; Fig. 2*E*,*F*; Table 2). Therefore, our data suggested that the blood glucose concentration rather than the circadian phase during which stroke occurred exacerbates stroke severity.

**Table 2:** Group comparisons analysis of data from this study

		*N*	Mean ± SEM	*p* value
Figure 2	One-way ANOVA followed by Tukey’s multiple comparisons test			
2*A*	ZT3–ZT9 SLEEP	13	165 ± 12	0.5226, daytime compared with nighttime
	ZT15–ZT21 saline	11	139 ± 11	
	ZT15–ZT21 glucose	13	302 ± 22	<0.0001, glucose compared with no glucose
2*B*	ZT3–ZT9 SLEEP	6	133 ± 14	0.5515, daytime compared with nighttime
	ZT15–ZT21 saline	6	101 ± 8	
	ZT15–ZT21 glucose	6	294 ± 33	<0.0001, glucose compared with no glucose
2*D*	ZT3–ZT9 SLEEP	11	7 ± 1	0.9851, daytime compared with nighttime
	ZT15–ZT21 saline	10	8 ± 1	
	ZT15–ZT21 glucose	10	22 ± 6	0.0352, glucose compared with no glucose
2*E*	ZT3–ZT9 SLEEP	11	6 ± 1	0.8985, daytime compared with nighttime
	ZT15–ZT21 saline	10	8 ± 1	
	ZT15–ZT21 glucose	10	23 ± 6	0.0116, glucose compared with no glucose
Figure 3	Unpaired *t* test			
3*A*	Glucose + saline	12	311 ± 17	0.0289
	Glucose + MK801	12	370 ± 19	
3*B*	Glucose + saline	11	14 ± 2	0.0363
	Glucose + MK801	11	23 ± 4	
3*C*	Glucose + saline	11	12 ± 1	0.0075
	Glucose + MK801	11	23 ± 4	
Figure 4	Two-way repeated-measures ANOVA matching blood glucose from the same animal, which was followed by Tukey’s multiple comparisons test			
Baseline	Saline	7	178 ± 12	>0.05, compared with saline group
	AP5	7	193 ± 10	
	MK801	7	191 ± 8	
	CPP	6	190 ± 17	
Post-NMDAR blocker	Saline	7	175 ± 9	
	AP5	7	192 ± 17	0.7902, compared with saline group
	MK801	7	223 ± 10	0.0163, compared with saline group
	CPP	6	195 ± 18	0.7456, compared with saline group
Postglucose	Saline	7	345 ± 9	
	AP5	7	334 ± 25	0.9746, compared with saline group
	MK801	7	434 ± 10	<0.0001, compared with saline group
	CPP	6	372 ± 11	0.2813, compared with saline group
Figure 5	Unpaired *t* test			
5*A*	Glucose + saline	19	299 ± 13	0.0005
	Glucose + ketamine	18	380 ± 17	
5*B*	Glucose + saline	16	8 ± 1	0.0446
	Glucose + ketamine	16	15 ± 3	
5*C*	Glucose + saline	16	9 ± 1	0.0217
	Glucose + ketamine	16	16 ± 3	
Figure 6	Unpaired *t* test			
6*A*	Diabetic + saline	9	290 ± 13	0.0001
	Diabetic + MK801	9	403 ± 18	
6*B*	Diabetic + saline	9	8 ± 2	0.0454
	Diabetic + MK801	9	18 ± 4	
6*C*	Diabetic + saline	9	8 ± 2	0.0469
	Diabetic + MK801	9	18 ± 4	

**Table 3:** Linear regression analysis of data from this study

Figures		*N*	Goodness of fit (*R*^2^)	*p* value (significance of correlation)
2*C*	ZT3–ZT9 SLEEP	11	0.04456	0.5332
	ZT15–ZT21 saline	10	0.1903	0.2075
	ZT15–ZT21 glucose	10	0.01200	0.7632
	Pooled	31	0.2004	0.0116
3*D*	Glucose + saline	11	0.05862	0.4732
	Glucose + MK801	11	0.03878	0.5616
	Pooled	22	0.01671	0.5664
5*D*	Glucose + saline	16	0.03069	0.5164
	Glucose + ketamine	16	0.004256	0.8103
	Pooled	32	0.02799	0.3601
6*D*	Diabetic + saline	9	0.0006287	0.9489
	Diabetic + MK801	9	0.1605	0.2853
	Pooled	18	0.04701	0.3875

### Effect of MK801 on stroke severity in hyperglycemic mice

**Figure 3.**
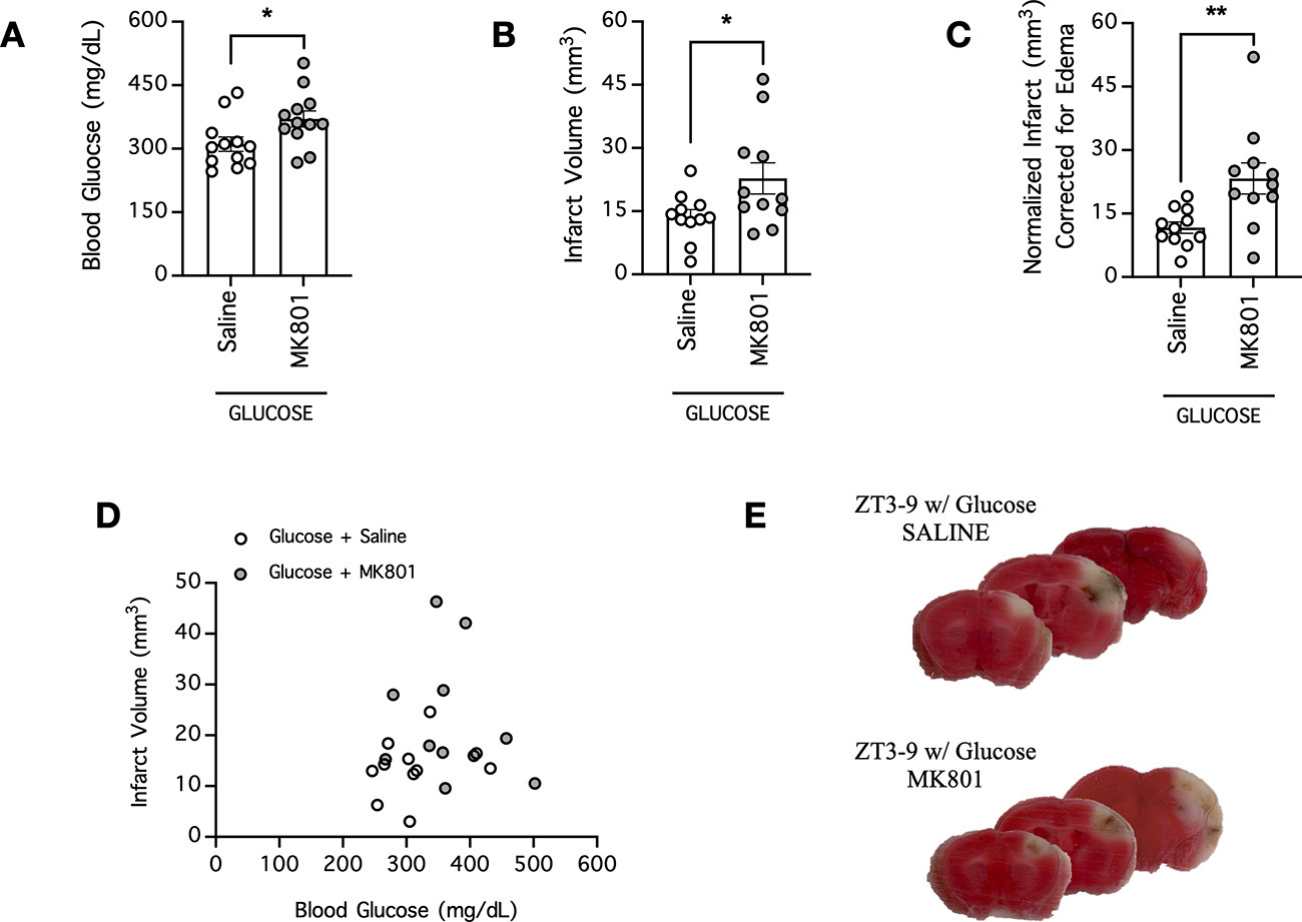
MK801 increases blood glucose and the ischemic infarction volume in hyperglycemic mice in which stroke was induced. ***A***, Blood was collected to measure the blood glucose concentrations of hyperglycemic mice pretreated with either saline or MK801 (4 mg/kg, i.p.) in the daytime group (ZT3–ZT9; sleep cycle for mice). Saline or MK801 was administered 60 min before blood collection, and mice were rendered hyperglycemic by a glucose injection (2.2 g/kg, i.p.) 20 min before blood collection; *n* = 12 per group. ***B***, The mice from ***A*** underwent cerebral ischemia induction by dMCAO immediately following blood collection, and the ischemic infarction volume was assessed 24 h postischemia; *n* = 11 per group. ***C***, Same as ***B***, except infarct was normalized to account for potential brain edema; *n* = 11 per group. In ***A–C***, *p < 0.05, **p < 0.01, Student’s *t* test. ***D***, XY plot of data from ***A***, ***B***. ***E***, Representative images of coronal brain sections of mice from ***B***. Functional brain tissue was stained red by TTC, and the infarct area remained pale white and unstained.

With our new analysis and data, we next asked whether a larger stroke infarction volume caused by hyperglycemia would be subject to neuroprotection by MK801 in a preclinical dMCAO stroke model that is otherwise well established to be resistant to neuroprotection by MK801 under normoglycemic conditions (Liu et al., 2020, 2021). Surprisingly, we found that the opposite was true (Fig. 3*A–E*; Table 2). To induce acute hyperglycemia, we infused mice with glucose 20 min before the induction of cerebral ischemia, and collected blood for analysis immediately before ischemia onset. Unexpectedly, pretreatment of these mice with MK801 60 min before ischemia onset further exacerbated their hyperglycemia (370 ± 19 mg/dl; *n* = 12; *p* = 0.0289, *t* test) compared with that of saline-treated hyperglycemic mice (311 ± 17 mg/dl; *n* = 12; Fig. 3*A*; Table 2). Consistent with the exacerbated hyperglycemia and the effect that blood glucose is known to have on stroke severity, MK801 also increased stroke infarction volume in hyperglycemic mice (23 ± 4 mm^3^; *n* = 11; *p* = 0.0363, *t* test) compared with that in hyperglycemic mice treated with saline (14 ± 2 mm^3^; *n* = 11; Fig. 3*B*,*E*; Table 2). When the infarct volumes were normalized to account for potential cerebral edema, MK801 increased normalized infarction volumes in hyperglycemic mice (23 ± 4 mm^3^; *n* = 11; *p* = 0.0075, *t* test) compared with those in hyperglycemic mice treated with saline (12 ± 1 mm^3^; *n* = 11; Fig. 3*C*,*E*; Table 2). Perhaps because of our small sample sizes, in contrast to expectations from clinical data (Riddle and Hart, 1982; Pulsinelli et al., 1983; Candelise et al., 1985; Berger and Hakim, 1986; Weir et al., 1997; Capes et al., 2001; Williams et al., 2002), we did not find significant correlation between blood glucose of individual mice and their infarct volumes (*p* > 0.05; Fig. 3*D*; Table 3). Taken together, our data demonstrated that hyperglycemia did not promote the neuroprotective effect of MK801 in the preclinical stroke model. Instead, they raised the intriguing possibility that one of the reasons for the clinical failure of NMDAR antagonism is because of a potential exacerbating effect of NMDAR antagonists on stroke outcome in hyperglycemic patients.

### Uncompetitive NMDAR antagonist increases blood glucose in normoglycemic mice

**Figure 4.**
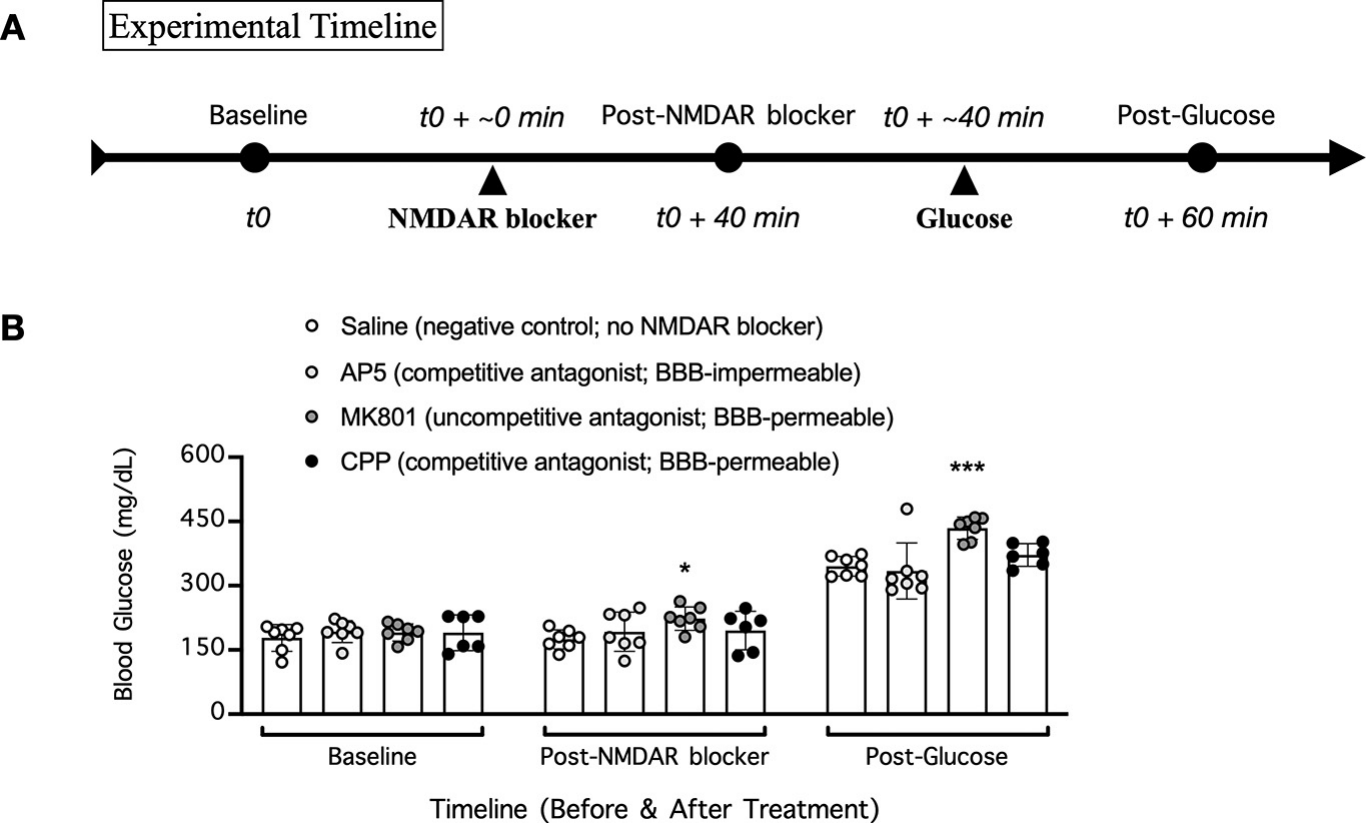
Uncompetitive but not competitive antagonists of the NMDAR increase blood glucose in normoglycemic and hyperglycemic mice. ***A***, Experimental timeline. Blood was collected before any treatments (baseline) and 40 min after injection of NMDAR antagonists: AP5 (10 mg/kg, i.p.), MK801 (4 mg/kg, i.p.), or CPP (10 mg/kg, i.p.); thereafter, the mice received a glucose injection (2.2 g/kg, i.p.) to induce hyperglycemia, and blood was once again collected 20 min after glucose injection. ***B***, Glucose concentrations of blood collected as described in ***A***; *n* = 6–7 per group; *p < 0.05, ***p < 0.001, one-way repeated-measures ANOVA, matching blood collected from the same mouse, followed by Tukey’s multiple comparisons test.

To examine whether MK801 would have a similar effect on blood glucose in normoglycemic mice and to identify the type of NMDAR antagonists that would have such an effect, we injected normoglycemic mice with three different types of NMDAR antagonists: (1) AP5, a competitive antagonist that is blood-brain barrier (BBB)-impermeable; (2) MK801, an uncompetitive antagonist that is BBB-permeable; and (3) CPP, a competitive antagonist that is BBB-permeable; we then collected blood 40 min postinjection to assess the effect of these drugs on blood glucose (Fig. 4*A*,*B*; Table 2). Blood glucose concentrations before injection were 178 ± 12 mg/dl (*n* = 7) in the saline group, 193 ± 10 mg/dl (*n* = 7) in the AP5 group, 191 ± 8 mg/dl (*n* = 7) in the MK801 group, and 190 ± 17 mg/dl (*n* = 6) in the CPP group; there was no significant difference between different groups (*p* > 0.05, Tukey’s multiple comparisons test; Fig. 4*B*). After injection of the respective antagonists in normoglycemic mice, blood glucose concentrations were 175 ± 9 mg/dl (*n* = 7) in the saline group, 192 ± 17 mg/dl (*n* = 7) in the AP5 group, 223 ± 10 mg/dl (*n* = 7) in the MK801 group, and 195 ± 18 mg/dl (*n* = 6) in the CPP group; importantly, MK801, but not AP5 (*p* = 0.7902, compared with saline group, Tukey’s multiple comparisons test) or CPP (*p* = 0.7456), significantly increased blood glucose in normoglycemic mice (*p* = 0.0163; Fig. 4*B*). To examine whether different types of NMDAR antagonists would similarly influence blood glucose in a hyperglycemic state, the above mice were further infused with glucose, and their blood was collected 20 min later to measure blood glucose concentrations (Fig. 4*A*,*B*; Table 2). The blood glucose concentrations after glucose infusion were 345 ± 9 mg/dl (*n* = 7) in the saline group, 334 ± 25 mg/dl (*n* = 7) in the AP5 group, 434 ± 10 mg/dl (*n* = 7) in the MK801 group, and 372 ± 11 mg/dl (*n* = 6) in the CPP group; therefore, MK801, but not AP5 (*p* = 0.9746, compared with saline group, Tukey’s multiple comparisons test) or CPP (*p* = 0.2813), significantly increased blood glucose in hyperglycemic mice (*p* < 0.0001; Fig. 4*B*). Thus, our data demonstrate that an uncompetitive NMDAR antagonist such as MK801 increases blood glucose in both normoglycemic and hyperglycemic mice. In contrast, competitive antagonists of NMDARs have no effect on blood glucose in either normoglycemic or hyperglycemic mice.

### Ketamine increases blood glucose and exacerbates stroke infarct in hyperglycemic mice

**Figure 5.**
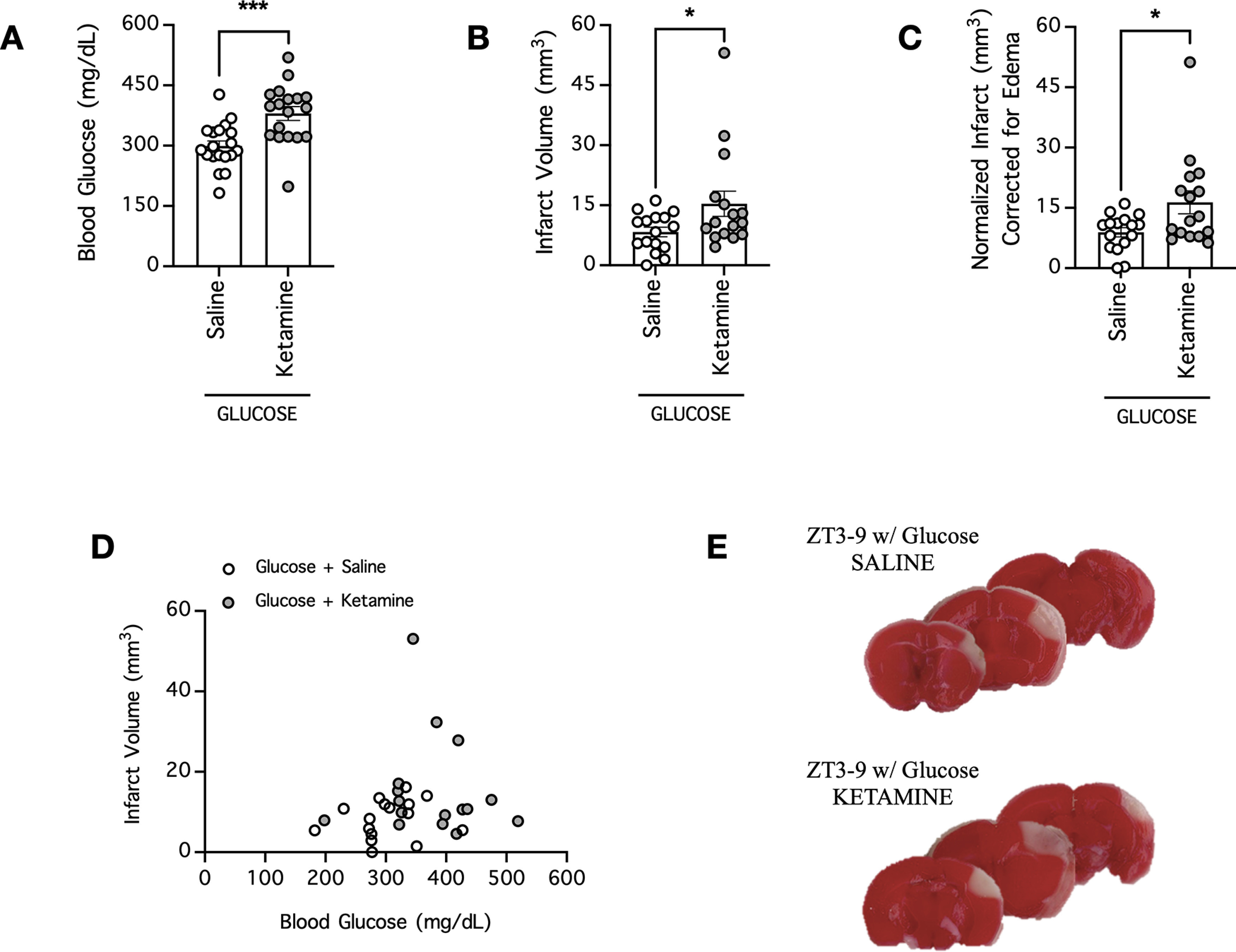
Ketamine increases blood glucose and the ischemic infarction volume in hyperglycemic mice subjected to stroke. ***A***, Blood was collected to measure the blood glucose concentrations of hyperglycemic mice pretreated with either saline or ketamine (10 mg/kg, i.p.) in the daytime group (ZT3–ZT9; sleep cycle for mice). Saline or ketamine was administered 60 min before blood collection, and mice were rendered hyperglycemic by a glucose injection (2.2 g/kg, i.p.) 20 min before blood collection; *n* = 18–19 per group. ***B***, The mice from ***A*** underwent cerebral ischemia induction by dMCAO immediately following blood collection, and the ischemic infarction volume was determined 24 h postischemia; *n* = 16 per group. ***C***, Same as ***B***, except infarct was normalized to account for potential brain edema; *n* = 16 per group. In ***A–C***, *p < 0.05, ***p < 0.001, Student’s *t* test. ***D***, XY plot of data from ***A***, ***B***. ***E***, Representative images of coronal brain sections of mice from ***B***. Functional brain tissue was stained red by TTC, and the infarct area remained pale white and unstained.

To validate that the effect of MK801 on blood glucose and stroke severity was because of its pharmacological property as an NMDAR uncompetitive antagonist, we tested whether ketamine, another NMDAR uncompetitive antagonist, would have similar effects. Mice were injected with ketamine 60 min before ischemia onset and then injected with glucose 20 min before ischemia onset to achieve a hyperglycemic state; thereafter, blood was collected to measure the blood glucose concentration immediately before ischemia onset. Ketamine strongly increased the blood glucose concentration of these mice to 380 ± 17 mg/dl (*n* = 18) compared with saline-treated mice, which had a blood glucose concentration of 299 ± 13 mg/dl (*n* = 19; *p* = 0.0005, *t* test; Fig. 5*A*; Table 2). In addition, 24 h after induction of cerebral ischemia by dMCAO, ketamine-treated mice had a larger ischemic infarction of 15 ± 3 mm^3^ (*n* = 16) than saline-treated mice, which had an ischemic infarction of 8 ± 1 mm^3^ (*n* = 16; *p* = 0.0446, *t* test; Fig. 5*B*,*E*; Table 2). When the infarct volume were normalized to account for potential cerebral edema, ketamine-treated mice had a larger normalized infarction of 16 ± 3 mm^3^ (*n* = 16) than saline-treated mice, which had an normalized infarction of 9 ± 1 mm^3^ (*n* = 16; *p* = 0.0217, *t* test; Fig. 5*C*,*E*; Table 2). Nevertheless, with our small sample sizes, we did not find significant correlation between blood glucose of individual mice and their infarct volumes (*p* > 0.05; Fig. 5*D*; Table 3). Therefore, similar to MK801, ketamine exacerbates hyperglycemia in mice and worsens stroke outcome.

### MK801 increases blood glucose and exacerbates stroke infarct in diabetic mice

**Figure 6.**
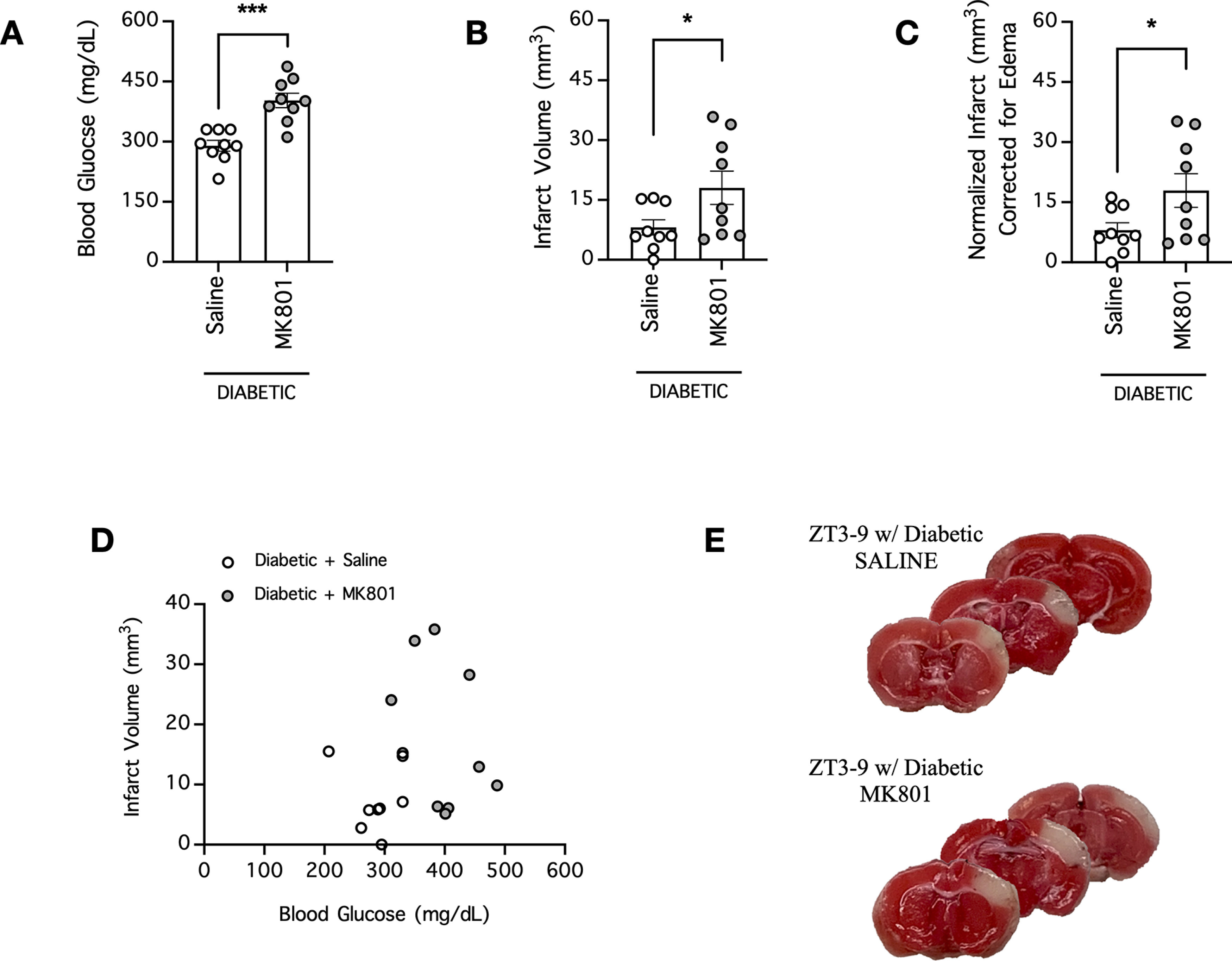
MK801 increases blood glucose and the ischemic infarction volume in diabetic mice subjected to stroke. ***A***, Mice were subjected to repeated STZ injections initiated two weeks before cerebral ischemia to induce diabetes mellitus. Blood was collected to measure the blood glucose concentrations of diabetic mice 60 min after pretreatment with either saline or MK801 (4 mg/kg, i.p.) in the daytime group (ZT3–ZT9; sleep cycle for mice); *n* = 9 per group. ***B***, The mice from ***A*** underwent cerebral ischemia induction by dMCAO immediately following blood collection, and the ischemic infarction volume was determined 24 h postischemia; *n* = 9 per group. ***C***, Same as ***B***, except infarct was normalized to account for potential brain edema; *n* = 9 per group. In ***A–C***, *p < 0.05, ***p < 0.001, Student’s *t* test. ***D***, XY plot of data from ***A***, ***B***. ***E***, Representative images of coronal brain sections of mice from ***B***. Functional brain tissue was stained red by TTC, and the infarct area remained pale white and unstained.

To better address whether the effect of NMDAR uncompetitive antagonist on blood glucose and stroke severity might be clinically relevant, we tested whether MK801 would have a similar effect in a mouse model of diabetes mellitus. Mice were rendered diabetic by STZ injections more than a week before the experiment (STZ-resistant mice were excluded; Table 4), and were injected with MK801 60 min before cerebral ischemia by dMCAO. When blood was collected for analysis immediately before ischemia onset, MK801 increased the blood glucose concentration of these diabetic mice to 403 ± 18 mg/dl (*n* = 9) compared with saline-treated diabetic mice, which had a blood glucose concentration of 290 ± 13 mg/dl (*n* = 9; *p* = 0.0001, *t* test; Fig. 6*A*; Table 2). Consistent with the exacerbated hyperglycemia, MK801-treated diabetic mice had a larger ischemic infarction of 18 ± 4 mm^3^ (*n* = 9) than saline-treated diabetic mice, which had an ischemic infarction of 8 ± 2 mm^3^ (*n* = 9; *p* = 0.0454, *t* test; Fig. 6*B*,*E*; Table 2). When the infarct volume were normalized to account for potential cerebral edema, MK801-treated diabetic mice had a larger normalized infarction of 18 ± 4 mm^3^ (*n* = 9) than saline-treated diabetic mice, which had a normalized infarction of 8 ± 2 mm^3^ (*n* = 9; *p* = 0.0469, *t* test; Fig. 6*C*,*E*; Table 2). However, with our small sample sizes, we did not find significant correlation between blood glucose of individual mice and their infarct volumes (*p* > 0.05; Fig. 6*D*; Table 3). Taken together, our data showed that MK801 exacerbates hyperglycemia in diabetic mice and worsens stroke outcome.

**Table 4:** Mice excluded from cerebral infarct analysis

Figures		*N*	Reason for exclusion	Blood glucose concentrations
2	ZT3–ZT9 SLEEP	2	1 × Died during surgery 1 × Died after reperfusion	168, 135
	ZT15–ZT21 saline	1	1 × Died after reperfusion	187
	ZT15–ZT21 glucose	3	2 × Died during surgery 1 × Died after reperfusion	238, 306, 301
3	Glucose + saline	1	1 × Died during surgery	279
	Glucose + MK801	1	1 × Died during surgery	379
5	Glucose + saline	3	2 × Died during surgery 1 × Died after reperfusion	287, 229, 332
	Glucose + ketamine	2	2 × Died during surgery	403, 415
6	STZ treatment	4	Not diabetic	186, 172, 188, 165
Total		17		

## Discussion

NMDARs are a widely sought-after therapeutic target for the development of stroke treatments (Lai et al., 2014). Decades of experimental evidence have suggested that during stroke, a dramatic increase in extracellular glutamate causes excessive stimulation of NMDARs; consequently, opening of the NMDAR channel pore leads to calcium ion influx into ischemic neurons. Calcium ions then trigger the release of nitrogen and oxygen free radicals, as well as other neuronal death-signaling cascades, to cause stroke injury. Therefore, drugs that inhibit NMDARs or the NMDAR-mediated release of free radicals have been tested in preclinical animal models in the hope that they will be beneficial to stroke patients in the clinic. These include competitive antagonists, such as AP5 and CPP, which directly bind to glutamate-binding sites on NMDARs; uncompetitive antagonists, such as MK801 and ketamine, which block the NMDAR channel pore when the receptors are activated such that calcium influx cannot occur even when the receptors are activated; and drugs that interfere with the NMDAR-mediated release of free radicals, such as NA-1 or α-phenyl-butyl-tert-nitrone, which do not inhibit NMDARs directly but interfere with NMDAR-mediated neuronal injury. Although these drugs are neuroprotective in many animal models of stroke, none have proven to be successful in stroke clinical trials (Lai et al., 2014; Hill et al., 2020).

Several reasons why NMDAR antagonists failed clinical trials have been postulated and have been extensively reviewed by our laboratory (Lai et al., 2014). Notably, these drugs are neuroprotective in only some animal models of cerebral ischemia (McDonald et al., 1987; Ozyurt et al., 1988; Park et al., 1988a, b; Ikonomidou et al., 1989; Olney et al., 1989; Rod and Auer, 1989; Rod et al., 1990; Swan and Meldrum, 1990; Gill et al., 1991; Buchan et al., 1992; Hatfield et al., 1992; Lo et al., 1994; Margaill et al., 1996; Ma et al., 1998) and not in others (Buchan et al., 1991; LeBlanc et al., 1991; Nellgård et al., 1991; Nellgård and Wieloch, 1992; Liu et al., 2020, 2021). Even in the same animal model of cerebral ischemia, the neuroprotective efficacy of NMDAR antagonists might be contingent on the specific experimental condition or condition of the animal during the experiment, such as their body temperature (Ikonomidou et al., 1989; Buchan and Pulsinelli, 1990; Corbett et al., 1990; Warner et al., 1991; Memezawa et al., 1995; Alkan et al., 2001; Gerriets et al., 2003). In the present study, we further demonstrate that uncompetitive NMDAR antagonists, including MK801 and ketamine, can exacerbate stroke outcome by increasing blood glucose in hyperglycemic mice. Therefore, given the high prevalence of hyperglycemia among stroke patients (Scott et al., 1999; Williams et al., 2002), we recommend that future clinical trials testing uncompetitive NMDAR antagonists preemptively measure blood glucose concentrations and exclude stroke patients who are moderately hyperglycemic from participating in the trial. Such a measure would maximize clinical trial success and avoid unnecessary risk for stroke patients participating in the trial.

The mechanism by which blood glucose exacerbates stroke outcome seems to be multifactorial and involves an increased likelihood of ischemia-induced seizure (Siemkowicz and Hansen, 1978), an impairment of postischemic cerebral blood flow (Ginsberg et al., 1980), augmented lactic acidosis (Welsh et al., 1980), and worsened ischemia-induced BBB disruption (Dietrich et al., 1993). In addition to smaller mammals (Siemkowicz and Hansen, 1978; Siemkowicz, 1981; Pulsinelli et al., 1982; de Courten-Myers et al., 1988, 1994; Yip et al., 1991), juvenile monkeys exhibited exacerbated ischemic brain damage following glucose administration (Myers and Yamaguchi, 1977), and blood glucose concentrations have consistently been correlated with worse stroke outcome in the clinic (Riddle and Hart, 1982; Pulsinelli et al., 1983; Candelise et al., 1985; Berger and Hakim, 1986; Weir et al., 1997; Capes et al., 2001; Williams et al., 2002). Notably, although an acute increase in blood glucose can exacerbate stroke severity, therapeutically decreasing blood glucose does not necessarily improve outcome in animals subjected to cerebral ischemia (Siemkowicz and Hansen, 1978; de Courten-Myers et al., 1994) or in stroke patients in clinical trials (Gray et al., 2007; Johnston et al., 2019).

Recently, one prominent study reported that the circadian phase during which stroke occurs can explain the translational failure of neuroprotective agents, including MK801 and α-phenyl-butyl-tert-nitrone (Esposito et al., 2020). The authors found that mice that had ischemic stroke during their sleep cycle (daytime for mice; nighttime for humans) had larger cerebral infarctions and benefited from neuroprotective agents, whereas mice that had ischemic stroke during their wake cycle had a smaller infarction and these drugs failed to show any neuroprotection. Because clinical trials typically enroll patients who had a stroke during the daytime (human wake cycle), the findings of this study might explain the translational failure of neuroprotective agents (Esposito et al., 2020). In this study, mice subjected to cerebral ischemia induced by dMCAO had similar infarct volume regardless of the circadian phase during which stroke occurs. Moreover, when stroke infarct was increased by induction of hyperglycemia, NMDAR uncompetitive antagonists like MK801 or ketamine did not offer neuroprotection; rather, these drugs increased ischemic infarction volumes by exacerbating preexisting hyperglycemia. One caveat in our study is that we experimented with only one model of cerebral ischemia and used only male mice, and therefore, we cannot exclude the possibility that the circadian phase during which stroke occurs can have an effect in female, in other models of cerebral ischemia, including the models used in the previous circadian study (Esposito et al., 2020), or in other physiological and comorbid states. Nevertheless, our data show conclusively that cerebral ischemia occurring in the daytime do not necessarily translate into larger infarction.

Because of the differences in the mechanism by which they antagonize NMDARs, competitive antagonists such as AP5 and CPP and uncompetitive antagonists such as MK801 and ketamine are known to exert different overall pharmacological effects (Willetts et al., 1990). For example, AP5 but not MK801 inhibits temporary memory storage in a delay-dependent manner (Tonkiss and Rawlins, 1991). Likewise, AP5 but not MK801 inhibits glutamate-dependent metabotropic NMDAR functions, including low-frequency stimulation and β-amyloid-induced synaptic depression (Kessels et al., 2013; Nabavi et al., 2013) and NMDAR-mediated dendritic blebbing and delayed inward current (Weilinger et al., 2016). Moreover, competitive but not uncompetitive antagonists of NMDARs block glycine-mediated neuroprotection *in vitro* and *in vivo* (Hu et al., 2016; Chen et al., 2017). On the other hand, uncompetitive but not competitive antagonists of NMDARs are effective in increasing brain glucose and dopamine metabolism (Boddeke et al., 1992; Bubser et al., 1992) and are liable to become substances of abuse in accordance with self-administration studies in animals (Willetts et al., 1990). Unfortunately, in most cases, the exact reason why different classes of antagonists confer distinct pharmacological effects is not completely understood. In this study, we further showed that uncompetitive but not competitive antagonists of NMDARs increase blood glucose concentrations in normoglycemic and hyperglycemic mice, which could help partly explain the clinical failure of this class of NMDAR antagonists in stroke trials. The hyperglycemic effect of uncompetitive antagonists could also partly explain why only this class of NMDAR blockers can increase brain glucose metabolism.

In conclusion, we showed in this study that the circadian phase during which stroke occurs does not necessarily affect ischemic infarction. Moreover, uncompetitive antagonists of NMDARs, including MK801 and ketamine, increase blood glucose in normoglycemic and hyperglycemic mice, and this can result in exacerbated stroke outcome in hyperglycemic mice. Given that hyperglycemia is present in ∼60–80% of stroke patients, our finding likely explains the translational failure of this class of NMDAR antagonists from bench to clinic.

## Synthesis

Reviewing Editor: Lindsay De Biase, University of California Los Angeles Decisions are customarily a result of the Reviewing Editor and the peer reviewers coming together and discussing their recommendations until a consensus is reached. When revisions are invited, a fact-based synthesis statement explaining their decision and outlining what is needed to prepare a revision will be listed below. The following reviewer(s) agreed to reveal their identity: Amy Gleichman.

In this manuscript, the authors examine how stroke size and susceptibility to MK801-induced neuroprotection are influenced by sleep state and blood glucose level. This manuscript is in direct response to a prominent paper published last year that suggests significant circadian effects on stroke size and response to neuroprotective interventions. This is a somewhat brief report with limited findings. However, given the prominence of the original paper that is being challenged here, both reviewers agree that a short but rapid report to challenge some potentially mis-interpreted results is warranted. NMDARs are known to play a central role in excitotoxic neuronal cell death caused by ischemic stroke. In general, blood glucose/hyperglycemia has been increasingly recognized as an insufficiently investigated variable in stroke trials and the reasons for NMDAR antagonist failure in stroke trials continue to be ambiguous. This manuscript may potentially offer some new insights.

However, both reviewers also agree that some of the methods and results require clarification.

Major comments:

1. The results shown are interesting, and potentially quite impactful as clinical trial designs strive to take into account variables like stroke timing and hyperglycemic state. However, there are some aspects of experiment design in the present manuscript that make it difficult to directly compare their data to the Esposito et al data that are being challenged.

1a. The authors show in figure 4 that MK-801 alone changes blood glucose level, and the effect is even more pronounced in the context of hyperglycemia. In figure 3 they also show that MK-801 elevates blood glucose in hyperglycemic mice. Therefore, it doesn’t seem entirely appropriate that they pool the neuroprotection (NMDA antagonist) and control data from Esposito et al when looking for differences in blood glucose across circadian phases. In the Esposito supplementary data on glucose, it also looks like the day/night difference is much larger in the neuroprotection-treated animals, so pooling the two in the analysis in this paper is a bit misleading. The authors state this was necessary for statistical power - but the authors should be more transparent that the data were handled in this way. They can first show neuroprotection and control data that is not pooled and then present the pooled data, labeling it as such. This way, readers can clearly see what the effect of the NMDA antagonist is on the structure of the data.

1b. It is also a bit confusing that the authors attempt to replicate the Esposito et al results by checking pre-stroke glucose levels (Fig. 2 and 3). The Esposito et al levels reported are entirely from post-stroke timepoints, so it isn’t fully valid to directly compare them. This doesn’t necessarily invalidate their results - the lack of difference in stroke size between the day and night animals (fig 2B) is critical here, regardless of the timing of the glucose measurements. However, when trying to replicate another group’s work and challenge their conclusions, using mismatched datapoints like this makes a direct comparison more complicated. Did the authors perform any blood collection and analysis of glucose levels post-stroke? Incorporating these into the manuscript would allow a much more complete comparison of the implications of the present results compared to the Esposito et al results. If these types of additional experiments are not added, the authors need to be more restrained in their conclusions - articulate their re-interpretation and conclusions of new work, while pointing out the strengths and weaknesses of their and Esposito’s approach.

2. It would be helpful to have more detail on how the authors measured infarct volume, particularly given that it is the main outcome measure in their study. Did they take edema into account? If so, by which method? If not, why not?

3. This study appears to be limited to all-male adult mice of a defined age group, and a single preclinical stroke model - distal, permanent (dMCAo) with occlusion period of 2 hr. Yet factors other than hyperglycemia are known to affect stroke outcome/size (e.g., gender, other physiological and comorbid states). Testing the impact of hyperglycemia and NMDAR antagonism in another model of stroke or in female mice would greatly strengthen the manuscript, especially given the prominence of the study they are challenging. If they authors are not able to add these experiments, the limitations of the present study need to be more thoroughly discussed (including limitation to male mice and a specific stroke model) and they need to be much more restrained in their critique of the Esposito study.

4. While the present study investigates effects of acute hyperglycemia (via analysis of glucose vs. vehicle treatments) in a rodent model, it is not clear how findings associated with acute hyperglycemia would be translatable. NMDAR antagonists also have to be administered either before or shortly after stroke onset to be effective. If the authors could select one NMDAR antagonist (e.g., MK801) and add datasets to show effects of hyperglycemia severity (mild-moderate-severe) during acute interval (20 min prior to stroke onset) this may somewhat enhance the findings. Ideally, if data from a chronic hyperglycemic model (e.g., diabetic/prediabetic mice) could be added, this would also greatly strengthen the study and be more immediately relevant to human patient populations.

5. Are there correlations between blood glucose levels of individual mice and the resulting infarct volume in their study. This can be analyzed via linear regression. Especially in mice that have unusually large infarct volume (Fig. 2B, 3B, 5B) - what was the blood glucose of these specific mice?

6. Although the figure legends say that mice were subjected to infarct after blood collection, the number of data points for infarct volume does not match the number of data points for blood glucose levels. This is the case in Fig 2, 3, and 5. Presumably this is related to the small % of failed surgeries they mention in the methods. However, can they clarify that these drop out data points were evenly / randomly distributed across their experiment groups? Can they clarify that these animals for which there are no infarct volume measurements were not at the top or bottom of their experiment group in terms of blood glucose measurements? The authors need to be fully transparent about any data points that were excluded and why.

Minor points:

1. The authors state that analysis of Esposito data at the 24hr time point was not analyzed “because the source data were inconsistent with the published data shown in the data table; thus it was unclear which blood glucose data were the correct version.” Esposito et al. could be consulted for comment and/or clarification regarding the source data and validity of this re-analysis.

2. It is a bit bizarre to have a timeline schematic (Fig 4) with no times listed on it. Times are found in the text and figure legend, but would be useful on the schematic itself.

3. The timing of NMDAR antagonist delivery in relation to stroke onset is not totally clear in the text nor figures. It is described in the figure legends, but should also be very clearly described in the results text and the methods.

4. In the methods section, the authors state that “[mice were] kept normothermic by means of an autoregulated heating pad with continuous feedback from a rectal temperature probe”. This reviewer is wondering whether or not any other variables were specifically monitored and/or corrected for? Either way, it would be important to clarify this.

5. For the methods section: some more details are needed in the description of the stroke procedure [or citation to previously published methods for their model]
